# The role of interventional cardiac magnetic resonance (iCMR) in a typical atrial flutter ablation: The shortest path may not always be the fastest

**DOI:** 10.1016/j.ijcha.2022.101078

**Published:** 2022-06-28

**Authors:** Geertruida P. Bijvoet, Robert J. Holtackers, Hedwig M.J.M. Nies, Casper Mihl, Sevasti-Maria Chaldoupi

**Affiliations:** a Cardiovascular Research Institute Maastricht (CARIM), Maastricht University, Maastricht, the Netherlands; b Department of Cardiology, Maastricht University Medical Center, Maastricht, the Netherlands; c Department of Radiology and Nuclear Medicine, Maastricht University Medical Center, Maastricht, the Netherlands

## 1 Introduction

Cardiac magnetic resonance (CMR)-guided RF ablation emerged as a novel innovative technique to treat cavo-tricuspid isthmus (CTI) dependent atrial flutter. With this interventional CMR (iCMR) technique, the electrophysiological procedure is performed while the patient is inside the CMR scanner. Advantages of iCMR include real-time imaging of the heart, real-time tracking and navigation of the catheters [1], [2], differentiation of normal and abnormal myocardium (e.g. scar from myocardial infarction and oedema formation in ablation lesions), visualization of the location and extent of the acute ablation lesion, as well as assessment of potential complications [1]. In addition, no harmful X-ray radiation is required which benefits both patient as and healthcare personnel.

## 2 Case report

A 65-year-old male with symptomatic persistent typical atrial flutter was referred for rhythm management to our centre. Previous ECGs confirmed a typical counterclockwise atrial flutter. His past medical history includes hypertension, gout, sleep apnoea, and epilepsy. In the past few years, more than three electrical cardioversions with early recurrences of atrial flutter were performed. Amiodaron to maintain sinus rhythm was initiated.

At the time of presentation in the outpatient clinic, the ECG showed sinus rhythm with a rate of fifty beats per minute. Physical examination was unremarkable. Echocardiography showed a good left ventricular function without valvular dysfunction and moderate left atrial dilatation (39 ml/m2). Laboratory analysis was unremarkable. The patient had no contraindications for magnetic resonance imaging; and after the patient provided informed consent, he was scheduled for an iCMR procedure.

In our institution, iCMR procedures are performed using a conventional 1.5 T MRI scanner (Ingenia; Philips Healthcare, Best, the Netherlands) that can be transformed into an interventional CMR suite.[3] The time-out procedure, general anaesthesia, sterile draping, and echo-guided vascular access to the femoral vein were performed outside the iCMR suite. The patient was in sinus rhythm and transferred to the iCMR suite and inserted into the scanner.

Pre-ablation imaging commenced with a baseline ECG-triggered respiratory-navigated 3D whole-heart balanced steady-state free precession (bSSFP) sequence to acquire the full anatomy of the heart and surrounding structures. The heart was segmented to create a 3D shell which was then integrated into the navigation system (iSuite; Philips Healthcare, Best, the Netherlands) as a roadmap for the iCMR procedure and active catheter tracking. In contrast to passive catheter tracking that relies on catheter visualization due to susceptibility artefacts, active tracking uses micro coils within the catheter tip to determine its location. The CTI was visualized in three imaging planes. In addition to the right anterior oblique (RAO) and the left anterior oblique (LAO) view, we also used a transversal view to visualize the isthmus from superior. The RAO view shows the isthmus in its full length while the LAO view reveals the medial or lateral position of the CTI line regarding the intra-atrial septum and the ostium of the coronary sinus (CS).

Following baseline imaging, MRI compatible catheters (Vision-MR Ablation Catheter; Imricor Medical Systems, Inc. Burnsville, MN, USA) were introduced in the femoral vein. These unique catheters regard two bipole irrigated-tip ablation catheters with two MR receive coils in the distal end for active MR tracking, displaying its real-time location inside the 3D shell (**see video online**). One catheter was placed in the CS, followed by the ablation catheter. Although these catheters are 9F, their maneuverability and stability are comparable with irrigated ablation catheters using in fluoroscopic-guided ablation procedures. Prior to ablation, a ‘design line’ was created to identify the optimal route through the isthmus towards the inferior caval vein, followed by the definitive ablation line.

**Fig. 1:**
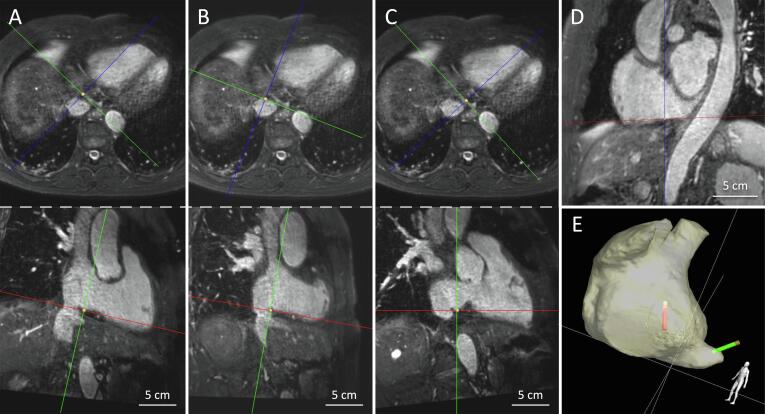
Panels A, B, and C show three potential locations of the CTI ablation line in the transversal view (top row) and the RAO view (bottom row). Panel D shows the LAO view. Panel E shows the 3D anatomical shell.

The optimal target ablation line is different for each patient, based on the route that gives the best catheter stability while taking into consideration the presence of a prominent sub-eustachian pouch or Eustachian ridge, and the length and thickness of the isthmus. In this patient, iCMR provided unique information about these anatomic variations that may challenge the CTI ablation. The MRI images showed that a lateral isthmus was interference with the hepatic vein, which would challenge catheter stability. A more septal isthmus position was rather thick with potentially increasing difficulties to create transmural ablation lesions. Finally, a medial position of the CTI was chosen (Fig. 1). Cine MRI at this location indicated a rather long isthmus (22 mm end-diastolic and 35 mm end-systolic), with a normal ridge thickness of 3 mm. In total, 23 ablation overlapping lesions (mean power (SD) 35.74 (1.17) W, mean temperature (SD) 37.67 (5.12) C^o^ and mean duration(SD) 44 (17) sec of ablation lesions) were required to complete the CTI line and create a bidirectional block (Fig. 2A**, right panels**). This bidirectional block was confirmed by a color-coded map representing the time delay between the pacing signal of the CS catheter and the activation time of the areas around the tricuspid annulus (Fig. 2A**, left panel**). During a 30-minute waiting period, additional ECG-triggered breath-hold black-blood T2-weighted MR imaging was performed to visualize edema in the ablation region (Fig. 2B). These images clearly show edema at the location of the CTI ablation line. The entire procedure was performed successful with no signs of pericardial effusion or valvular damage, as confirmed by CMR imaging just before removing the catheters.

**Fig. 2A:**
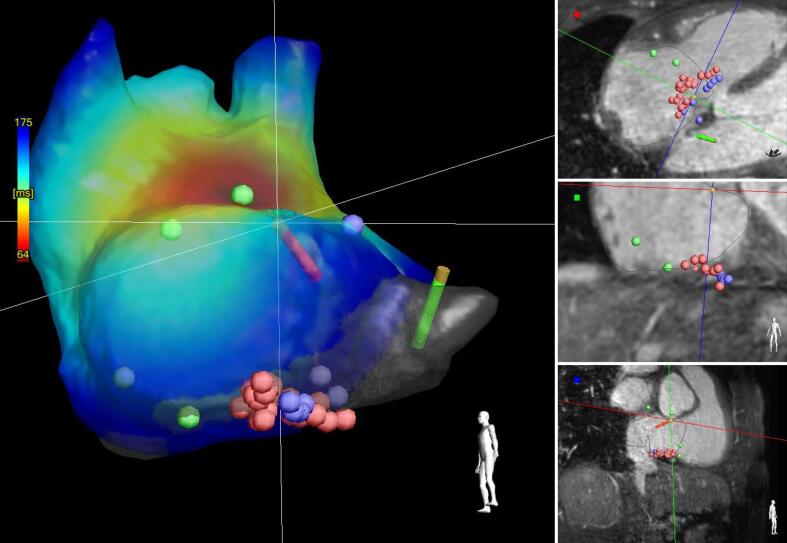
Right panels: Visualization of the ‘design line’ (purple dots) and the final ablation lesions (red dots) in three different imaging views. Left panel: Electrical anatomical mapping (EAM) acquired directly following ablation, visualizing the activation times throughout the right atrium with reference to the pacing signal from the diagnostic catheter in the coronary isthmus. Blue = longest time interval at the most lateral position along the tricuspid annulus, red = shortest time interval at the areas nearby the intra-atrial septum and the ostium of the coronary sinus. (For interpretation of the references to color in this figure legend, the reader is referred to the web version of this article.)

**Fig. 2B:**
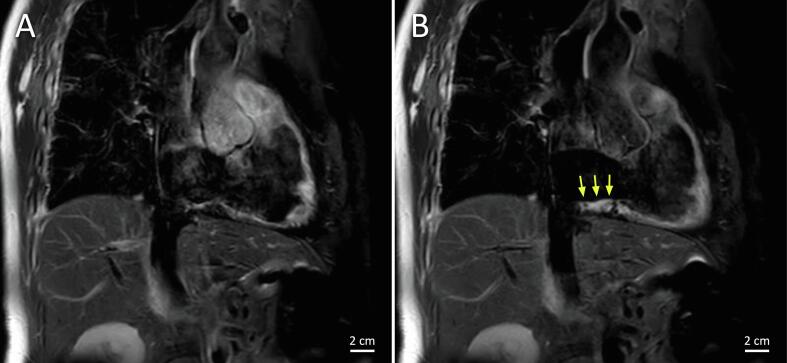
T2-weighted MR images of the CTI in the RAO view before (A) and after (B) ablation showing edema in the ablation lesions, indicated by the yellow arrows.

## 3 Conclusion

In this case we illustrate that iCMR allows the visualization of patient specific anatomy of the CTI to guide catheter ablation from the tricuspid annulus to the inferior caval vein. The CMR-derived anatomical information was used to decide on a relatively long medial ablation line in a rather thin-walled region of the CTI, which also allowed a better catheter stability than a more lateral position. Further studies are required to evaluate whether real-time visualization of the complete ablation line anatomy, real-time tracking of the ablation catheters, and electrical confirmation of a bidirectional block increase procedural success rate.

## Declaration of Competing Interest

The authors declare that they have no known competing financial interests or personal relationships that could have appeared to influence the work reported in this paper.

## References

[1] Chubb H., Harrison J.L., Weiss S., Krueger S., Koken P., Bloch L.O. (2017). Development, preclinical validation, and clinical
translation of a cardiac magnetic resonance - electrophysiology
system with active catheter tracking for ablation of cardiac
arrhythmia. JACC Clin. Electrophysiol..

[2] Paetsch I., Sommer P., Jahnke C., Hilbert S., Loebe S., Schoene K., Oebel S., Krueger S., Weiss S., Smink J., Lloyd T., Hindricks G. (2019). Clinical workflow and applicability of
electrophysiological cardiovascular magnetic resonance-guided
radiofrequency ablation of isthmus-dependent atrial
flutter. Eur. Heart J. Cardiovasc.
Imaging.

[3] Bijvoet G.P., Holtackers R.J., Smink J., Lloyd T., Hombergh C.L.M., Debie L.J.B.M., Wildberger J.E., Vernooy K., Mihl C., Chaldoupi S.-M. (2021). Transforming a pre-existing MRI environment into an
interventional cardiac MRI suite. J. Cardiovasc. Electrophysiol..

